# Cyclophosphamide for anticancer therapy-induced interstitial lung disease in the modern era: a retrospective cohort study

**DOI:** 10.3389/fonc.2025.1567317

**Published:** 2025-05-16

**Authors:** Honami Katahara, Kaede Baba, Hiromichi Nakajima, Chikako Funasaka, Chihiro Kondoh, Yoichi Naito, Hibiki Udagawa, Kohei Shitara, Tomoaki Sasaki, Toshikatsu Kawasaki, Toru Mukohara

**Affiliations:** ^1^ Department of Pharmacy, National Cancer Center Hospital East, Kashiwa, Japan; ^2^ Department of Medical Oncology, National Cancer Center Hospital East, Kashiwa, Japan; ^3^ Department of Experimental Therapeutics, National Cancer Center Hospital East, Kashiwa, Japan; ^4^ Department of General Internal Medicine, National Cancer Center Hospital East, Kashiwa, Japan; ^5^ Department of Thoracic Oncology, National Cancer Center Hospital East, Kashiwa, Japan; ^6^ Department of Gastroenterology and Gastrointestinal Oncology, National Cancer Center Hospital East, Kashiwa, Japan; ^7^ Department of Diagnostic Radiology, National Cancer Center Hospital East, Kashiwa, Japan

**Keywords:** drug-induced interstitial lung disease, cyclophosphamide, corticosteroid-refractory, immune-related pneumonitis, immunosuppressant

## Abstract

**Background:**

Drug-induced interstitial lung disease (DIILD) is a serious complication of cancer treatment that is primarily treated with corticosteroids. However, effective standardized regimens for corticosteroid-refractory DIILD have not been established. Cyclophosphamide (CPA) is an immunosuppressant that is potentially effective against DIILD, but supporting evidence is limited, particularly for diseases induced by novel chemotherapeutic drugs. In this study, we examined the efficacy and safety of CPA in corticosteroid-refractory DIILD caused by various anticancer drugs.

**Methods:**

We retrospectively reviewed the medical records of patients who underwent CPA therapy for corticosteroid-refractory DIILD at the National Cancer Center Hospital East between January 2013 and October 2023. Corticosteroid-refractory DIILD was defined as cases of DIILD classified as grade ≥3 according to the Common Terminology Criteria for Adverse Events (CTCAE) version 5.0, in which no improvement was observed within 48 hours after initiating corticosteroid therapy. The primary endpoint was 30-day survival post-CPA. The secondary endpoints included radiological improvements and changes in oxygen supplementation.

**Results:**

Fifteen patients (median age 73 years; 80% male) were included in the analysis. Patients were classified into molecular-targeted drugs (MT; 20%, 3/15), MT + cytotoxic drugs (33%, 5/15), immune checkpoint inhibitors (ICI) ± cytotoxic drugs (27%, 4/15), and cytotoxic drugs alone (20%, 3/15) groups. The overall 30-day survival rate was 47% (7/15). Improvement of oxygen demand allowed 20% (3/15) of patients to discontinue oxygen supplementation. CPA demonstrated drug class-dependent efficacy: highest in the MT group (67% survival, 2/3), less benefit in the cytotoxic drugs alone group (0% survival, 0/3). Adverse events included grade 3 anemia (n=2), grade 4 neutropenia (n=1), and grade 2 cytomegalovirus infection (n=1), with no treatment-related deaths.

**Conclusion:**

CPA exhibited potential efficacy for corticosteroid-refractory DIILD, particularly in patients with MT-induced DIILD, with manageable toxicity. The differential responses based on drug category suggest tailored approaches to DIILD management may be warranted. These findings may contribute to optimizing the management of severe DIILD during cancer treatment.

## 1 Introduction

Interstitial lung diseases (ILDs) are a heterogeneous group of disorders that affect pulmonary parenchyma and exhibit various clinical, radiological, and pathological features. Drug-induced interstitial lung disease (DIILD) is an ILD subtype that occurs as a result of exposure to certain drugs. Anticancer drugs are a major cause of DIILDs (23–51% of cases), followed by antirheumatic agents and antibiotics (1). DIILD is one of the most serious complications of anticancer treatment (2) and is associated with treatment-related deaths (3). The pathogenic mechanisms underlying DIILD remain incompletely defined; however, its development is primarily attributed to two principal mechanisms: direct cytotoxic injury to lung tissue and dysregulated immune-mediated damage (4). These processes can result in inflammation, fibrosis, and pulmonary scarring, particularly in severe cases.

Historically, a variety of cytotoxic agents, including bleomycin, gemcitabine, and irinotecan, have been reported to be the major causes of DIILD, with high incident rates ranging from 10% to 20% (5). However, with the development of molecular-targeted therapies (MT) and immunotherapies, the number of causative anticancer agents has increased. Notably, the HER2-targeted antibody-drug conjugate, trastuzumab deruxtecan (T-DXd) as well as immune checkpoint inhibitors (ICI) are effective against a variety of cancer types and are commonly used in clinical practice (6, 7); however, a high incidence of ILD and related deaths has been observed (8, 9). Other drugs, such as epidermal growth factor receptor (EGFR) tyrosine kinase inhibitors for lung cancer and cyclin-dependent kinase 4 and 6 (CDK4/6) inhibitors for breast cancer demonstrate high efficacy (10, 11), but they are also associated with DIILD (12, 13). This highlights the ongoing issue of high DIILD incidence and mortality rates associated with novel agents, which underscores the need for more effective management strategies for DIILD.

The management of DIILD involves the discontinuation of causative drug therapy and the initiation of immunosuppressive agents, depending on the severity of the symptoms (14). Although evidence supporting the benefit of corticosteroids is largely observational (15), their use for DIILD is widely accepted. However, some patients exhibit resistance to corticosteroid therapy. When no clinical improvement is observed after administering high-dose corticosteroids (prednisone 1–2 mg/kg/day) within 48 hours, the condition is defined as corticosteroid-refractory DIILD (14, 16). For patients with corticosteroid-refractory DIILD, alternative immunosuppressive agents, such as azathioprine, cyclophosphamide (CPA), mycophenolate mofetil, and infliximab, should be considered. All of these agents have been evaluated in studies in the context of corticosteroid-refractory, nonidiopathic pulmonary fibrosis (17). In particular, CPA, which acts as an immunosuppressant by alkylating DNA and inhibiting the growth of fast-dividing immune cells, such as T- and B-lymphocytes (18), improves pulmonary function in patients with scleroderma-related ILD or interstitial pneumonia with autoimmune features (IPAF) (17, 19). Given that the pathogenesis of DIILD likely involves immune-mediated mechanisms, the anti-inflammatory and antifibrotic properties of CPA may be beneficial in controlling disease progression. However, clinical evidence regarding the use of CPA specifically in corticosteroid-refractory DIILD remains limited (20).

Corticosteroid-refractory DIILD is associated with a particularly poor prognosis (21), evidence-based treatment strategies for this disease are urgently needed. Therefore, we conducted a retrospective analysis to explore the efficacy and safety of CPA for corticosteroid-refractory DIILD in a clinical setting.

## 2 Materials and methods

### 2.1 Patients and data collection

This retrospective cohort study was conducted and reported in accordance with the Strengthening the Reporting of Observational Studies in Epidemiology (STROBE) guidelines (22, **Supplementary Table 1**). The medical records of patients treated with CPA for corticosteroid-refractory DIILD at the National Cancer Center Hospital East (NCCHE). Patients were eligible for inclusion if: 1) treated at the NCCHE between January 2013 and October 2023; 2) DIILD diagnosis via multidisciplinary assessment (clinical/radiologica30l); 3) had corticosteroid-refractory DIILD, defined as cases classified as grade ≥3 according to the Common Terminology Criteria for Adverse Events (CTCAE) version 5.0 that showed no clinical or radiological improvement within 48 hours after initiating high-dose corticosteroid therapy (prednisone ≥1 mg/kg/day) (14, 16); 4) received CPA for corticosteroid-refractory DIILD; and 5) sufficient data available. Patients were excluded if: 1) concurrently enrolled in an interventional clinical trial at the time of CPA administration, or 2) a confirmed active infection as the primary cause of the respiratory condition. Patient demographics and disease characteristics were collected at the time of CPA administration. These included age, gender, primary tumor, performance status (Eastern Cooperative Oncology Group, ECOG), smoking index, radiation history, and the number of prior treatments. The causative drugs, imaging patterns of DIILD, and oxygen supplementation amount were also collected. The approval for the study and waiver for patient informed consent were received from the Institutional Review Board (IRB) of the National Cancer Center (IRB number 2023-308), in accordance with the principles stated in Japan’s Ethics Guidelines for Epidemiological Research.

### 2.2 Assessment

#### 2.2.1 Severity of the DIILD

DIILD was diagnosed based on laboratory and imaging findings through a multidisciplinary discussion involving the attending physician, radiologist, and pulmonologist. To classify grade ≥3 DIILD according to CTCAE more objectively, severity in this study was categorized based on the levels of respiratory support required, reflecting the degree of hypoxemia and respiratory compromise. Five severity groups were established: I) no oxygen supplementation; II) nasal cannula oxygenation; III) oxymask (OM) or Oxymizer^®^; IV) reservoir mask (RM); or V) high flow nasal cannula (HFNC) or noninvasive positive pressure ventilation (NIPPV; including two-stage or continuous positive pressure). This classification system provides an objective, clinically relevant assessment of DIILD severity that correlates with increasing impairment of gas exchange and allows for standardized comparison across patients.

#### 2.2.2 Imaging pattern of the DIILD

The imaging patterns of DIILD were categorized as follows: i) acute respiratory distress syndrome (ARDS)/diffuse alveolar damage (DAD); ii) nonspecific interstitial pneumonia (NSIP); iii) organizing pneumonia (OP); iv) acute eosinophilic pneumonia; and v) hypersensitivity pneumonitis (HP) revised by radiologist (T.S) according to previous reports (23–25).

#### 2.2.3 Efficacy of cyclophosphamide on DIILD

The primary endpoint was survival rate after 30 days of CPA administration. The secondary endpoint was improvement detected by clinical and imaging response. Clinical response was defined by alterations in oxygen supplementation and was evaluated pre- and 30 days post-CPA administration. Overall survival following CPA administration was estimated using the Kaplan-Meier method. The efficacy of CPA based on imaging response was evaluated by radiologist (T.S.) who was blinded to the patients’ clinical outcomes. The assessment was performed by comparing baseline and follow-up imaging patterns to determine radiological improvement or progression. Baseline imaging was defined as the imaging patterns obtained at the time of cancer diagnosis. Based on the causative drugs, the patients were categorized into MT, MT + cytotoxic, ICI ± cytotoxic, and cytotoxic drugs alone groups. Statistical analyses were not performed because of the small sample size and retrospective nature of the study. Instead, descriptive analyses were conducted to provide an overview of the data.

#### 2.2.4 Adverse event assessment

Adverse events related to CPA administration were evaluated and graded according to the Common Terminology Criteria for Adverse Events (CTCAE) version 5.0.

## 3 Results

### 3.1 Patients

We identified 15 patients with corticosteroid-refractory DIILD who underwent treatment with CPA (**Table 1**, **Supplementary Figure 1**). The median age at diagnosis was 73 years (range: 43–84 years), with males comprising 80% (12 patients) of the cohort. Among the patients, 80% (12 patients) were current or former smokers, with a mean smoking index (packs/year) of 40.7 (range: 11.25–62.5). The primary cancer types were gastric (40%, 6 patients), lung (20%, 3 patients), breast (13%, 2 patients), and others (27%, 4 patients). In addition, 20% (3 patients) of the patients had a history of chest radiotherapy.

**Table 1:** Patient characteristics.

No.	Age (yr), Sex Primary tumor	Baseline pulmonary findings	Smoking index (pack years)	Previous thoracic RT	Causative drugs	Drug classification	Radiographic patterns		Prior treatments for ILD	oxygen supplementation at the start of CPA	Initial treatment to CPA Administration (days)	Survival status after 30 days
							At the time of DIILD diagnosis	At the time of CPA administration				
#1	69, F Breast	None	Never	No	Everolimus	MT	ARDS/DAD	–	mPSL 1,000 mg 3 days	HFNC FiO_2_ 0.9	3	alive
#2	72, F Breast	Multiple lung metastases	Never	Yes	Palbociclib	MT	ARDS/DAD	–	mPSL 1,000 mg 3 days	HFNC FiO_2_ 0.9	4	alive
#3	79, F Peritoneum	None	Never	No	Olaparib	MT	ARDS/DAD	–	mPSL 1,000 mg 3 days	RM 15 L/min	6	alive
#4	76, M Lung	Emphysema, right upper lobe lung cancer	53	No	CBDCA, PTX, Pembrolizumab	ICI± Cytotoxic drugs	ARDS/DAD	ARDS/DAD	mPSL 1,000 mg 3 days	HFNC FiO_2_ 0.5	4	alive
#5	69, M Gastric	None	40	No	FOLFOX, Nivolumab	ICI± Cytotoxic drugs	ARDS/DAD	–	PSL 0.5mg/kg IFX 5 mg/kg	HFNC FiO_2_ 0.7	6	alive
#6	76, M Lung	Right lower lobe lung cancer	45	Yes	Durvalumab	ICI± Cytotoxic drugs	OP, Radiation pneumonitis	ARDS/DAD	PSL 0.5mg/kg	HFNC FiO_2_ 0.7	39	dead
#7	60, M Lung	Emphysema, left upper lobe lung cancer	45	No	Nivolumab	ICI± Cytotoxic drugs	OP	ARDS/DAD	mPSL 1,000 mg 3 days	OM 6 L/min	8	dead
#8	69, M Colon	Lung metastasis, right pulmonary fistula	48	No	FOLFOX, Panitumumab	MT+ Cytotoxic drugs	ARDS/DAD	–	mPSL 1,000 mg 3 days	RM 10 L/min	4	alive
#9	74, M Gastric	Mild emphysema, NSIP	11.2	No	T-DXd	MT+ Cytotoxic drugs	ARDS/DAD		mPSL 1,000 mg 3 days	HFNC FiO_2_ 0.4	7	alive
#10	82, M Gastric	None	20	No	PTX, Ramucirumab	MT+ Cytotoxic drugs	OP	ARDS/DAD	mPSL 1,000 mg 3 days	RM 8 L/min	10	dead
#11	55, M Gastric	None	54	No	nab-PTX, Ramucirumab	MT+ Cytotoxic drugs	NSIP	ARDS/DAD	mPSL 1,000 mg 3 days	RM 13 L/min	3	dead
#12	84, M Gastric	None	62.5	No	nab-PTX, Ramucirumab	MT+ Cytotoxic drugs	ARDS/DAD		mPSL 1,000 mg 3 days	HFNC FiO_2_ 0.7	6	dead
#13	43, M Seminoma	Mild emphysema	43	No	Bleomycin	Cytotoxic drugs	ARDS/DAD	–	mPSL 1,000 mg 3 days	NPPV FiO_2_ 0.8	11	dead
#14	77, M Esophagus	UIP	49	Yes	FOLFOX	Cytotoxic drugs	ARDS/DAD	–	mPSL 1,000 mg 5 days	OM 10 L/min	5	dead
#15	72, M Gastric	None	18	No	SOX	Cytotoxic drugs	OP	OP>ARDS/DAD	mPSL 1,000 mg 3 days	NIPPV FiO_2_ 0.8	3	dead

ARDS/DAD, acute respiratory distress syndrome/diffuse alveolar damage; CBDCA, carboplatin; CPA, cyclophosphamide; DIILD, drug-induced interstitial lung disease; FiO_2_, fraction of inspired oxygen; FOLFOX, folinic acid, fluorouracil, and oxaliplatin; HFNC, high-flow nasal cannula; ICI, immune checkpoint inhibitors; IFX, infliximab; mPSL, methylprednisolone; MT, molecular targeted therapies; nab-PTX, nab-paclitaxel; NIPPV, noninvasive positive pressure ventilation; NSIP, nonspecific interstitial pneumonia; OM, oxymask; OP, organizing pneumonia; PTX, paclitaxel; PS, performance status (Eastern Cooperative Oncology Group, ECOG); PSL, prednisolone; RM, reservoir mask; RT, irradiation; SOX, S-1 and oxaliplatin; T-DXd, trastuzumab deruxtecan; UIP, usual interstitial pneumonia.

At the initial diagnosis of DIILD, radiographic findings predominantly showed ARDS/DAD patterns (67%, 10 patients), followed by OP (27%, 4 patients) and NSIP patterns (7%, 1 patient).

Based on the DIILD-causative drugs, the patients were classified into the following four groups: MT (20%, 3 patients), MT + cytotoxic drugs (33%, 5 patients), ICI ± cytotoxic drugs (27%, 4 patients), and cytotoxic drugs alone (20%, 3 patients). DIILD occurred between 38 and 548 days (median: 83 days) after initial drug administration. All patients received high-dose corticosteroids (methylprednisolone 1,000 mg for 3–5 days) before CPA, and one patient also received infliximab (5 mg/kg). The patients did not show improvement within 48 hours after initiating corticosteroid therapy. The interval between the initial DIILD treatment and CPA administration ranged from 3 to 39 days (median: 6 days). CPA was administered as a single dose in all patients, with the majority receiving 500 mg/body and two receiving 500 mg/m², determined based on institutional practice and clinical judgment for these severe, refractory cases, considering patient condition and potential hematologic toxicity.

### 3.2 Clinical outcomes of CPA therapy for DIILD

**Figure 1:**
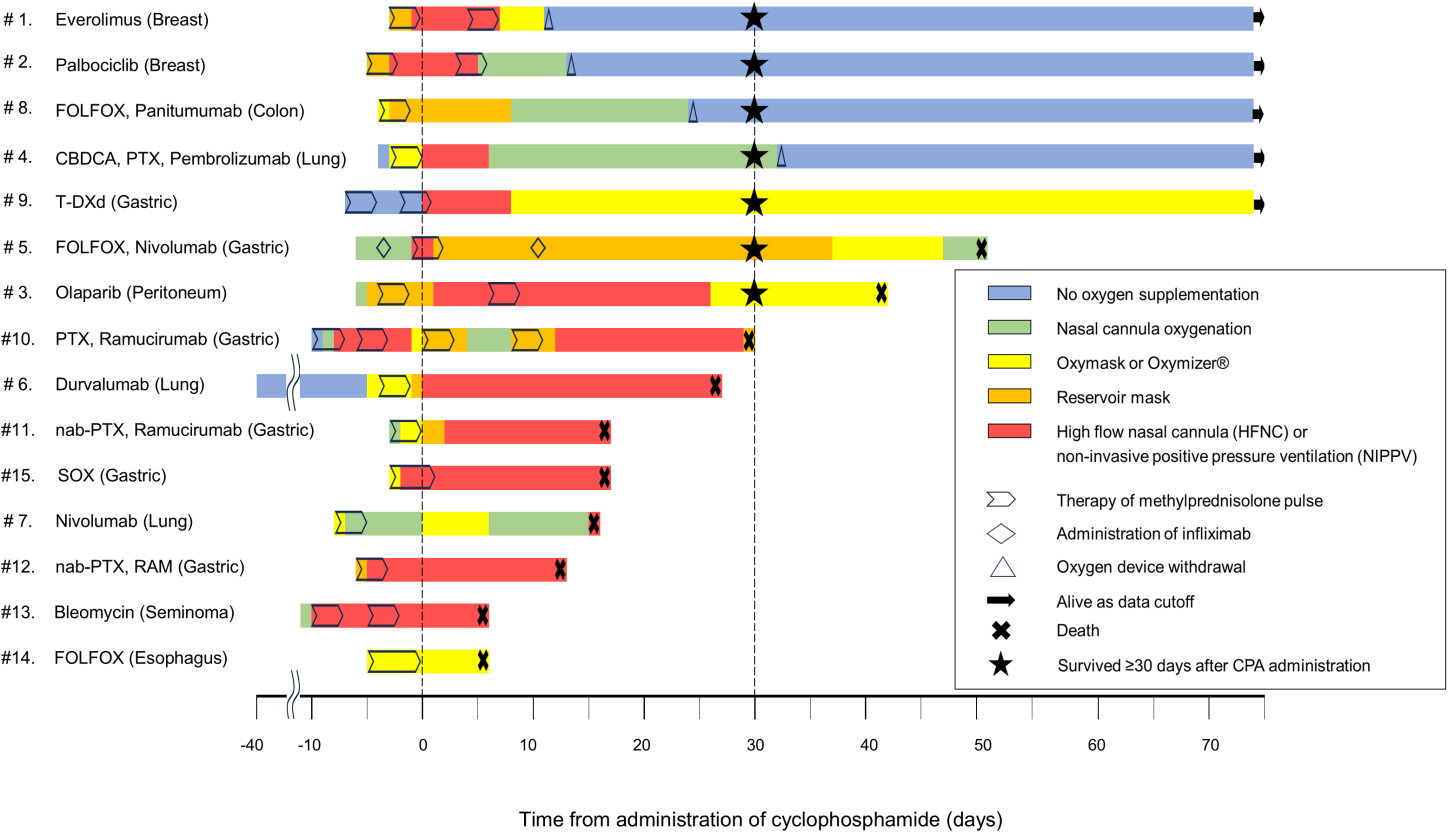
Swimmer plot showing changes in clinical outcomes and oxygen requirements in patients following CPA administration for DIILD. The swimmer plot demonstrates the clinical course of each patient following CPA administration, including the type and duration of oxygen supplementation, survival outcome, and key therapeutic interventions. The day of CPA administration was defined as day 0. Negative time points denote events before CPA administration, whereas the events following the administration are marked as positive. CBDCA, carboplatin; CPA, cyclophosphamide; DIILD, drug-induced interstitial lung disease; FOLFOX, folinic acid, fluorouracil, and oxaliplatin; HFNC, high-flow nasal cannula; nab-PTX, nab-paclitaxel; NIPPV, noninvasive positive pressure ventilation; PTX, paclitaxel; SOX, S-1 and oxaliplatin; T-DXd, trastuzumab deruxtecan.

**Figure 1** shows the dynamics of oxygen supplementation, treatment intervention, and the final survival outcomes following CPA administration to each patient.

#### 3.2.1 Survival status after 30 days

The 30-day survival rate post-CPA administration was 47% (7 patients). DILD was the cause of all the patient deaths within 30 days post-CPA administration. Among the seven surviving patients, five were discharged from the hospital, whereas two remained hospitalized and subsequently died on days 41 and 50 after CPA administration. **Supplementary Figure 2** shows the overall survival curve for the cohort.

#### 3.2.2 Oxygen supplementation

**Figure 2:**
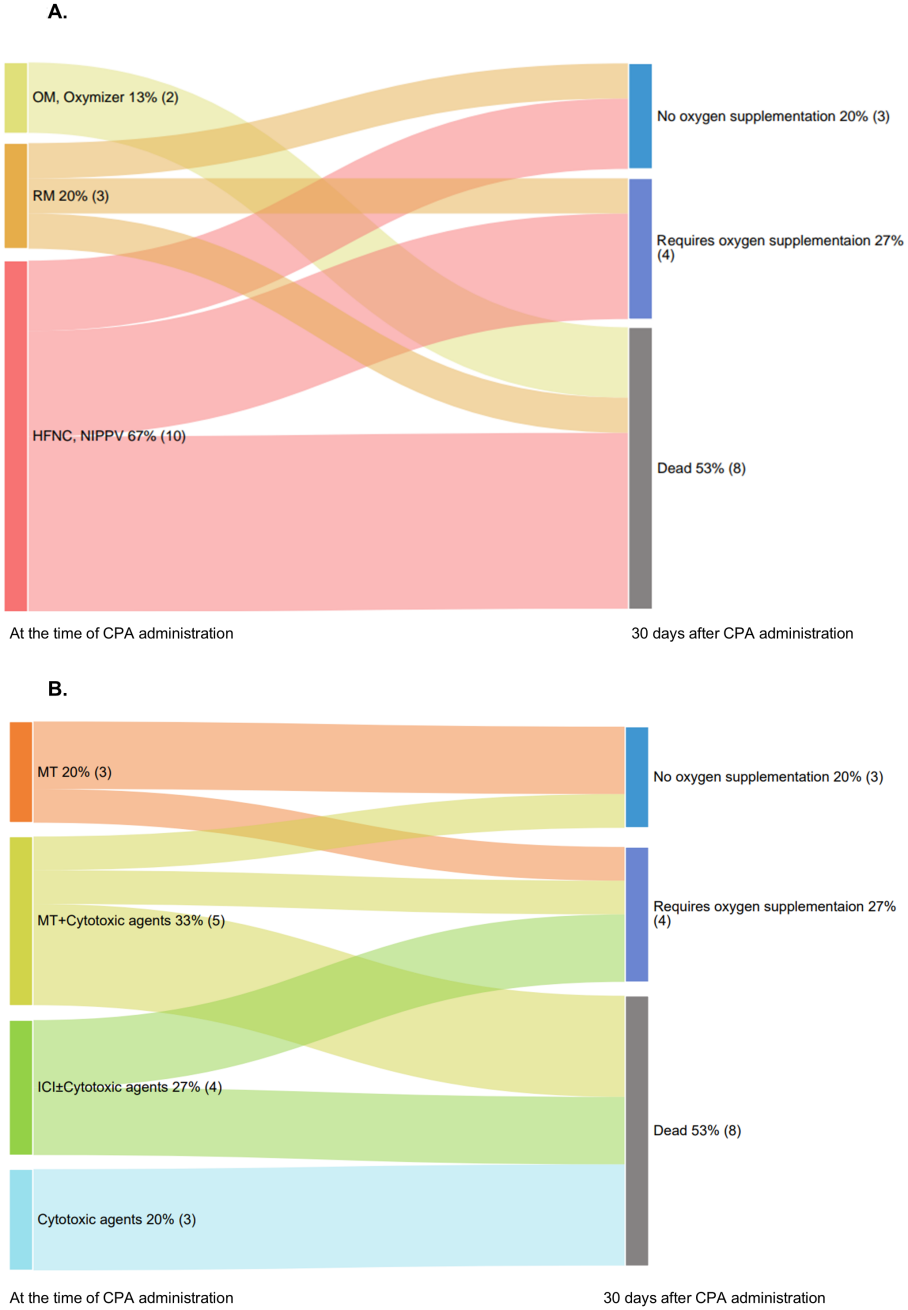
Oxygen supplementation status after CPA administration. **(A)** Sankey diagram illustrating the transition in oxygen supplementation status of patients at the time of CPA administration and 30 days later. **(B)** Sankey diagram illustrating patient outcomes 30 days after CPA administration and categorized by causative drugs. CPA, cyclophosphamide; HFNC, high-flow nasal cannula; ICI, immune checkpoint inhibitors; NIPPV, noninvasive positive pressure ventilation; OM, oxymask; RM, reservoir mask.

At the time of CPA administration, all patients required oxygen support to varying degrees. The highest oxygen supplementation during the period from the initiation of corticosteroid therapy to the start of CPA administration was as follows: OM or Oxymizer^®^ 13% (2 patients), RM 20% (3 patients), and HFNC or NIPPV 67% (10 patients). Subsequently, 20% (3 patients) of the patients no longer required any oxygen supplementation, whereas 27% (4 patients) continued to require some form of oxygen therapy, showing improvement. However, the remaining 53% (8 patients) did not experience any benefit (**Figure 2A**).

The outcomes at 30 days following CPA administration, categorized by the causative drugs, are shown in **Figure 2B**. In the MT group, 67% (2/3) no longer required oxygen supplementation, whereas 33% (1/3) still required it. In the MT + cytotoxic drugs group, 20% (1/5) experienced improvement without oxygen supplementation, whereas 80% (4/5) had different outcomes: one continued oxygen supplementation, and four died. In the ICI ± cytotoxic drugs group, 50% (2/4) of the patients continued requiring oxygen supplementation, whereas the other 50% (2/4) died. In the cytotoxic drugs alone group, all three patients died (**Figure 2B**).

#### 3.2.3 Imaging pattern

**Figure 3:**
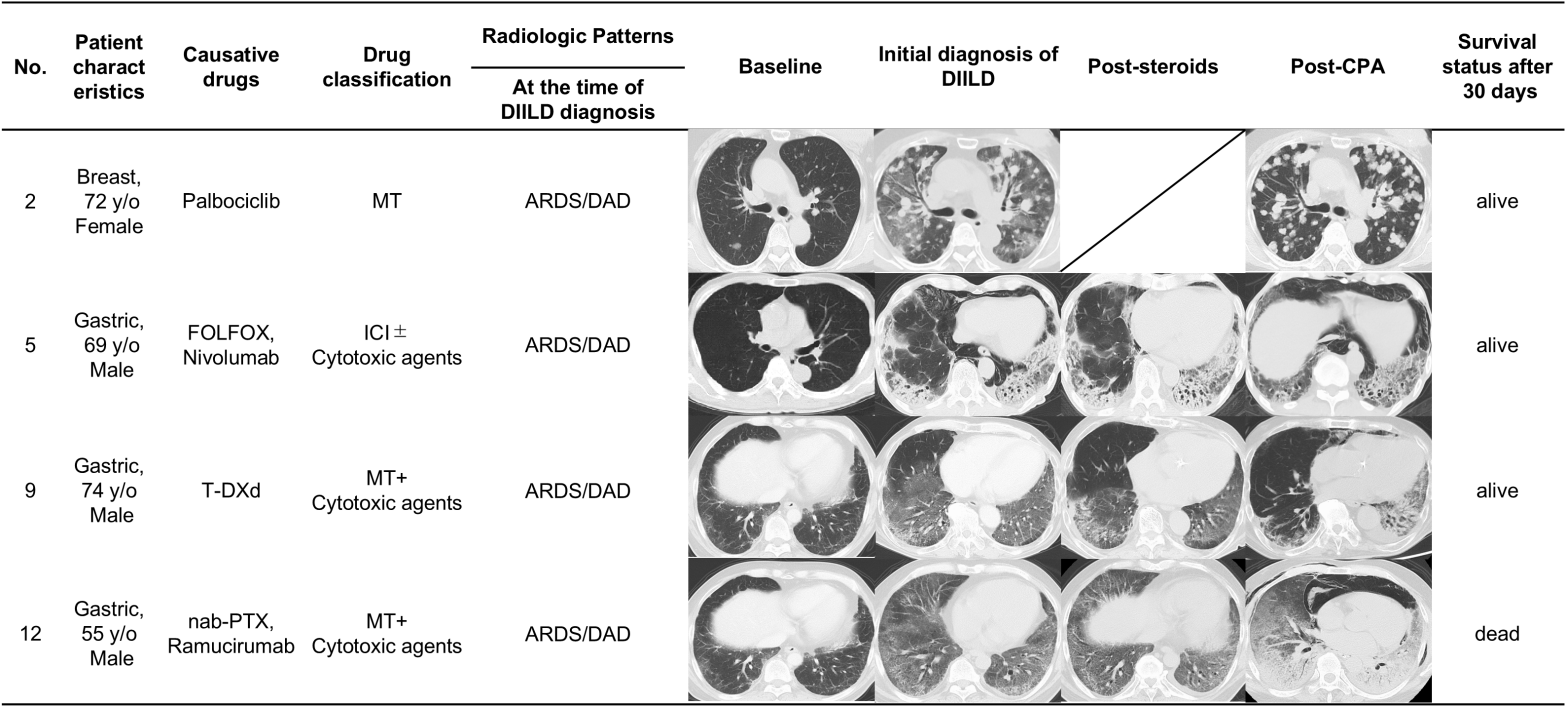
Serial radiologic imaging of patients with corticosteroid-refractory DIILD. Serial CT images showing radiologic patterns in four representative patients with corticosteroid-refractory DIILD who were treated with CPA. ARDS/DAD, acute respiratory distress syndrome/diffuse alveolar damage; CPA, cyclophosphamide; DIILD, drug-induced interstitial lung disease; FOLFOX, folinic acid, fluorouracil, and oxaliplatin; ICI, immune checkpoint inhibitors; MT, molecular-targeted therapies; NSIP, nonspecific interstitial pneumonia; OP, organizing pneumonia; PTX, paclitaxel; T-DXd, trastuzumab deruxtecan.

**Figure 3** shows a detailed comparison of sequential CT scans of patients, who developed DIILD, at four distinct time points: baseline, initial diagnosis of DIILD, post-corticosteroid treatment, and post-CPA administration. The CT images show the radiologic patterns, treatment response, and 30-day survival status of four patients with various cancer types treated with different causative agents.

- Case No. 2: A 72-year-old female patient with breast cancer treated with palbociclib in combination with endocrine therapy. At the time of DIILD diagnosis, the imaging findings revealed diffuse bilateral ground-glass opacities (GGO) consistent with ARDS/DAD pattern. Despite corticosteroid therapy, her oxygen demand increased from 10 L/min via RM to HFNC with an FiO_2_ of 90%. After CPA treatment, the GGO markedly decreased, and oxygen supplementation was discontinued within 13 days. The patient survived >30 days post-CPA administration and remained alive at the cutoff date.
- Case No. 5: A 69-year-old male patient with gastric cancer treated with FOLFOX and nivolumab. At DIILD diagnosis, imaging findings were consistent with ARDS/DAD pattern, showing diffuse bilateral GGO and patchy consolidation predominantly in lower lobes. Despite treatment with infliximab and corticosteroids, oxygen demand rose from 2 L/min via nasal cannula to HFNC with an FiO_2_ of 60%. After CPA administration, despite improvements in GGO but persistent consolidation and fibrotic changes, oxygen supplementation was maintained at 7 L/min via RM for 30 days following CPA administration. The patient survived 30 days, but ultimately died from DIILD after 50 days of CPA administration.
- Case No. 9: A 74-year-old male patient with gastric cancer treated with T-DXd with NSIP pattern at baseline. At the time of DIILD diagnosis, imaging showed ARDS/DAD pattern with diffuse bilateral GGO. Despite corticosteroid therapy, his oxygen needs increased to HFNC with an FiO_2_ of 40%. After CPA administration, oxygen supplementation was maintained at 5 L/min via Oxymizer^®^, with partial radiographic improvement but complications from cytomegalovirus infection and pneumothorax. The patient survived post-CPA administration, but died from pneumothorax 279 days later.
- Case No. 12: A 55-year-old male patient with gastric cancer received nab-paclitaxel and ramucirumab. At the time of DIILD diagnosis, imaging showed ARDS/DAD pattern with diffuse bilateral GGO. Despite corticosteroid therapy, the oxygen needs became as high as 10 L/min via RM to HFNC with a FiO_2_ of 70%. CPA administration resulted in minimal radiographic improvements and oxygen requirement was not improved. The patient unfortunately died 12 days after the CPA administration because of interstitial pneumonia.

### 3.3 Adverse events

Adverse events associated with CPA included grade 3 anemia in two patients, grade 4 neutropenia in 7% (1 patient), and grade 2 cytomegalovirus infection in 7% (1 patient). No deaths were attributed to adverse events.

## 4 Discussion

This study examined the effectiveness of CPA in 15 patients with DIILD caused by anticancer agents. To our knowledge, it is the first to report on the efficacy of CPA in DIILD induced by modern therapies, including MT and ICI. Although the efficacy of CPA in DIILD remains unclear, these real-world clinical data suggest that CPA may be an option for the treatment of corticosteroid-resistant DIILD provoked by modern anticancer drugs.

The development of DIILD is related to cytotoxicity and the immune responses which act independently or synergistically (4). The cytotoxic mechanisms include reactive oxygen species induced by drug exposure, such as bleomycin (26), impaired alveolar repair (27), reduced detoxification of pulmonary metabolites (28), and cytokine release (29). These mechanisms are frequently associated with cytotoxic anticancer drugs. In addition, T-DXd, an antibody-drug conjugate widely used to treat various cancers, is thought to be non-specifically taken up by alveolar macrophages via Fcγ receptors, releasing DXd within lung tissue, where it accumulates and causes alveolar damage (30). In contrast, immune-mediated mechanisms, including T-cell activation, dysregulation of regulatory T-cells (Tregs), and the inflammatory cytokine storm (31), often result in lung injury, particularly with ICIs (32), CDK4/6 inhibitors (33), mTOR inhibitors (34), and other MTs (35).

Our findings demonstrate drug category-dependent efficacy of CPA in DIILD treatment. This observed variable efficacy across drug categories can be explained by distinct pathophysiological mechanisms underlying DIILD development. For MT-induced DIILD (exemplified by CDK4/6 inhibitors, mTOR inhibitors, and PARP inhibitors), the pathogenesis primarily involves dysregulation of specific immune pathways, including T-cell activation and cytokine production. CPA’s potent suppression of T and B lymphocyte proliferation and function directly targets these mechanisms, potentially explaining the higher efficacy observed in this subgroup (18, 36). The established use of CPA in treating autoimmune disease and graft-versus-host diseases further supports its potential effectiveness against DIILD mediated by immune-related mechanisms (37).

In contrast, cytotoxic drug-induced DIILD (particularly with agents like bleomycin) involves direct cellular toxicity (the cytotoxic mechanisms), such as oxidative stress, alveolar epithelial cell death, and impaired tissue repair (21, 26), rather than immune-mediated inflammation, mechanisms that are largely independent of immune cell proliferation. This likely explains why all patients in the cytotoxic drugs alone group failed to respond to CPA therapy, suggesting that alternative approaches targeting antioxidant pathways or tissue repair mechanisms may be more appropriate for this subgroup.

In the ICI ± cytotoxic drugs group, the observed mixed response patterns (50% survival with continued oxygen requirements) likely reflect the complex immunopathology involving dual mechanisms: DIILD caused by immune-related mechanisms from ICIs and cytotoxic mechanisms from conventional agents. This mixed etiology may explain why CPA, with its primarily immunosuppressive mechanism of action, yielded only partial benefits in this subgroup. Furthermore, the poor outcomes in the two patients who developed DIILD after ICI monotherapy underscore the inherent challenges and severity of corticosteroid-refractory immune-related pneumonitis; however, conclusions based specifically on these two cases are limited. This observation aligns with previous studies investigating other immunosuppressants (e.g., infliximab, mycophenolate mofetil) for refractory ICI pneumonitis (20, 38, 39), which also reported variable effectiveness and often modest resolution rates. Our findings, along with case reports (20, 40), suggest that CPA could be considered an additional therapeutic option in this difficult refractory setting, although appropriate patient selection is likely critical.

This study has several limitations. First, it was from a single center and had a retrospective design, with a small sample size. Second, significant heterogeneity existed within the cohort regarding patient background factors, the specific anticancer drugs suspected of causing DIILD, and the timing of CPA administration. This heterogeneity complicates interpretation, particularly in attributing DIILD causality definitively to a single drug category when combination regimens involving multiple drug classes (e.g., MT + cytotoxic ICI + cytotoxic) were used. The possibility that the observed effects may result from a delayed response to corticosteroids cannot be ruled out. Third, the lack of a control group to compare CPA therapy with that of other immunosuppressive agents limited the assessment of the efficacy of CPA. Fourth, the predominantly male cohort (80%) limited our ability to explore potential sex-based differences. Fifth, the small number of patients receiving the 500 mg/m² CPA dose (n=2) compared to the 500 mg fixed dose (n=13) precluded any meaningful comparison of efficacy between these dosing strategies. A standardized, evidence-based dosing protocol for CPA does not currently exist for this specific clinical indication. Finally, bronchoalveolar lavage was not performed in these patients, making it difficult to definitively evaluate immune mechanisms and the role of CPA in immune modulation. Despite these limitations, this study provides valuable real-world insights into the use of CPA and highlights its potential role in managing immune-mediated DIILD. It also indicates that CPA may not be effective in treating corticosteroid-resistant DIILD caused by cytotoxic anticancer drugs. To further validate these findings, prospective studies with larger patient cohorts are needed.

In summary, this study suggests that, even in the current era of rapidly emerging anticancer therapies, CPA remains a promising option for managing corticosteroid-resistant DIILD caused by anticancer drugs. These real-world findings particularly highlight its potential efficacy in cases associated with MT. However, because of the variability in patient responses, further studies are needed to tailor treatment strategies according to the patient’s condition and the pathophysiology of DIILD.

## Data Availability

The datasets generated for this study are not publicly available but can be obtained from the corresponding author upon reasonable request.
