# Supplementary material for: Cyclophosphamide for anticancer therapy-induced interstitial lung disease in the modern era: a retrospective cohort study
Source: Front Oncol. 2025 May 16;15:1567317. doi: 10.3389/fonc.2025.1567317 (PMC12122744; doi:10.3389/fonc.2025.1567317)
Supplement: Supplementary file 1 [file DataSheet1.docx]

**Supplementary Table 1: STROBE checklist**

| **Item No** | **Topic** | **Recommendation** | **Location in Manuscript (Revised)** |
| --- | --- | --- | --- |
| TITLE and ABSTRACT |  |  |  |
| 1 | Title | (a) Indicate the study’s design with a commonly used term in the title or the abstract. | Title |
|  | Abstract | (b) Provide in the abstract an informative and balanced summary of what was done and what was found. | Abstract |
| INTRODUCTION |  |  |  |
| 2 | Background/ Rationale | Explain the scientific background and rationale for the investigation being reported. | Introduction |
| 3 | Objectives | State specific objectives, including any prespecified hypotheses. | Introduction |
| METHODS |  |  |  |
| 4 | Study design | Present key elements of study design early in the paper. | Methods (Section 2.1) |
| 5 | Setting | Describe the setting, locations, and relevant dates, including periods of recruitment, exposure, follow-up, and data collection. | Methods (Section 2.1) |
| 6 | Participants | (a) Give the eligibility criteria, and the sources and methods of selection of participants. Describe methods of follow-up. | Methods (Section 2.1), Supplementary Figure 1 |
|  |  | (b) For matched studies, give matching criteria and number of exposed and unexposed. | Not Applicable |
| 7 | Variables | Clearly define all outcomes, exposures, predictors, potential confounders, and effect modifiers. Give diagnostic criteria, if applicable. | Methods (Sections 2.1, 2.2), Table 1 |
| 8 | Data sources/ measurement | For each variable of interest, give sources of data and details of methods of assessment (measurement). Describe comparability of assessment methods if there is more than one group. | Methods (Sections 2.1, 2.2) |
| 9 | Bias | Describe any efforts to address potential sources of bias. | Methods (Section 2.1 - e.g., inclusion of all eligible patients), Discussion (Section 4 - Limitations) |
| 10 | Study size | Explain how the study size was arrived at. | Results (Section3.1 - stated N=15), Discussion (Section 4 - Limitations acknowledge small size) |
| 11 | Quantitative variables | Explain how quantitative variables were handled in the analyses. If applicable, describe which groupings were chosen and why. | Methods (Section 2.2.3 - e.g., Kaplan-Meier method), Results (Section 3.1 - Drug groups) |
| 12 | Statistical methods | (a) Describe all statistical methods, including those used to control for confounding. | Not Applicable due to small sample size  Discussion (Section 4 - Limitations) |
|  |  | (b) Describe any methods used to examine subgroups and interactions. | Methods (Section 2.2.3), Results (Section 3.2.2 - subgroup analysis by drug) |
|  |  | (c) Explain how missing data were addressed. | Not explicitly stated (Assume complete data for the 15 patients, or mention if imputation was used) |
|  |  | (d) If applicable, explain how loss to follow-up was addressed. | Not Applicable |
|  |  | (e) Describe any sensitivity analyses. | Not Applicable / Not Performed |
| RESULTS |  |  |  |
| 13 | Participants | (a) Report numbers of individuals at each stage of study—eg numbers potentially eligible, examined 9 for eligibility, confirmed eligible, included in the study, completing follow-up, and analysed. | Results (Section 3.1), Supplementary Figure 1 |
|  |  | (b) Give reasons for non-participation at each stage. | Supplementary Figure 1 |
|  |  | (c) Consider use of a flow diagram. | Supplementary Figure 1 |
| 14 | Descriptive data | (a) Give characteristics of study participants (eg demographic, clinical, social) and information on exposures and potential confounders. | Results (Section 3.1), Table 1 |
|  |  | (b) Indicate number of participants with missing data for each variable of interest. | Not explicitly stated (Assume complete data for the 15 patients, or mention if imputation was used) |
|  |  | (c) Summarise follow-up time (eg, average and total amount). | Figure 1 |
| 15 | Outcome data | Report numbers of outcome events or summary measures over time. | Results (Sections 3.2.1, 3.2.2), Figure 1, Supplementary Figure 2 |
| 16 | Main results | (a) Give unadjusted estimates and, if applicable, confounder-adjusted estimates and their precision (eg, 95% confidence interval). Make clear which confounders were adjusted for and why they were included. | Results (Sections 3.2.1, 3.2.2): Reports primary/secondary outcome rates (e.g., 30d survival 47%, O2 independence 20%). Confidence intervals and adjusted estimates are likely not presented due to the small sample size. |
|  |  | (b) Report category boundaries when continuous variables were categorized. | The main reported results (survival rate, oxygen improvement, descriptive subgroup outcomes) do not appear to involve the categorization of continuous variables for analysis purposes in this study. |
|  |  | (c) If relevant, consider translating estimates of relative risk into absolute risk for a meaningful time period. | Not Done |
| 17 | Other analyses | Report other analyses done—eg analyses of subgroups and interactions, and sensitivity analyses. | Results (Section 3.2.2 - Subgroup analysis by causative drug class), Methods (Section 2.2.3) |
| DISCUSSION |  |  |  |
| 18 | Key results | Summarise key results with reference to study objectives. | Discussion (Section 4, first and last para) |
| 19 | Limitations | Discuss limitations of the study, taking into account sources of potential bias or imprecision. Discuss both direction and magnitude of any potential bias. | Discussion (Section 4, Limitations para) |
| 20 | Interpretation | Give a cautious overall interpretation of results considering objectives, limitations, multiplicity of analyses, results from similar studies, and other relevant evidence. | Discussion (Section 4, entire) |
| 21 | Generalisability | Discuss the generalisability (external validity) of the study results. | Discussion (Section 4, Limitations paragraph implicitly addresses this via single center/small N) |
| OTHER INFORMATION |  |  |  |
| 22 | Funding | Give the source of funding and the role of the funders for the present study and, if applicable, for the original study on which the present article is based. | Funding Section (Section 7 - Stated none) |
|  | Ethics | State whether ethical approval was obtained and informed consent requirement was waived. | Ethical Statements (Section 9 - Stated IRB approval #2023-308, consent waived) |

**Supplementary Figures**

**Supplementary Figure 1: Patient Selection Flow Diagram**

Flow diagram illustrating the selection process for the study cohort.

Abbreviations: CPA, cyclophosphamide; DIILD, drug-induced interstitial lung disease; NCCHE, National Cancer Center Hospital East.

**Supplementary Figure 2: Survival after Administration of CPA**

Kaplan-Meier curve showing overall survival from CPA administration in the study cohort (n=15)

Abbreviations: CPA, cyclophosphamide.
